# The limbic system

**DOI:** 10.4103/0019-5545.33264

**Published:** 2007

**Authors:** V. Rajmohan, E. Mohandas

**Affiliations:** Elite Mission Hospital, Trichur, Kerala, India

“The hypothalamus, the anterior thalamic nucleus, the cingulate gyrus, the hippocampus and their interconnections, constitute a harmonious mechanism which may elaborate the functions of central emotion as well as participate in the emotional expression.”

**James Papez, 1937**

The limbic system consists of the phylogenetically old limbic lobe and other subcortical structures and their connections. Although not empirically proven, the limbic system is a functional concept which may be employed to explain various brain functions.[1]

## HISTORY

Paul Pierre Broca in 1878 spoke of ‘*le grand lobe limbique’* or the great limbic lobe and applied the term “limbic” (from the Latin *limbus* for border) to the curved rim of the cortex which incudes the cingulate and the parahippocampal gyri. However, its putative role in emotion was elaborated by the American physician, James Papez in 1937 in the seminal paper titled ‘A proposed mechanism of emotion’. This anatomical model is referred to as the Papez circuit.[2] Yakovlev in 1948 proposed Yakovlev's circuit in the control of emotion involving the orbitofrontal, insular and anterior temporal lobe cortex, the amygdala and the dorsomedial nucleus of thalamus.[3] In 1952, Paul D. MacLean coined the term “limbic system” to describe Broca's limbic lobe and related subcortical nuclei as the collective neural substrate for emotion.[1] MacLean was also instrumental in proposing and defining the Triune concept of the brain. MacLean's evolutionary “Triune brain theory” proposed that the human brain was in reality three brains in one: the R-complex (reptilian complex), the limbic system and the neocortex.[4] The concept of the limbic system has since been further expanded and developed by Nauta, Heimer and others.

## COMPONENTS OF THE LIMBIC SYSTEM

There is no universal agreement on the total list of structures, which comprise the limbic system. The brain regions that constitute the limbic system are:

1. Limbic cortex
  - Cingulate gyrus
  - Parahippocampal gyrus
2. Hippocampal formation
  - The dentate gyrus
  - Hippocampus
  - Subicular Complex
3. Amygdala
4. Septal area
5. Hypothalamus

These structures form a complex network for controlling emotion.[5]

### Limbic lobe

The limbic lobe situated at the inferomedial aspect of the cerebral hemispheres, consists of two concentric gyri surrounding the corpus callosum. Broca proposed that the larger outer gyrus be named “limbic gyrus” and the smaller inner one “the intralimbic gyrus”. The limbic gyrus (limbic lobe) consists of the isthmus of the cingulate gyrus, the parahippocampal gyrus (both of which are continuous via a bundle of white matter called “cingulum”) and the subcallosal area.[6]

The cingulate gyrus (Latin = Belt ridge) dorsal to the corpus callosum is heavily interconnected with the association areas of the cerebral cortex. The parahippocampal gyrus in the medial temporal lobe contains several distinct regions, the most important being the entorhinal cortex (ERC). The ERC funnels highly processed cortical information to the hippocampal formation and serves as its major output pathway.[5]

### The hippocampal formation

Hippocampal formation in the temporal lobe has three distinct zones:

1. The dentate gyrus
2. The hippocampus proper
3. The subiculum

Embryologically, the hippocampal formation is an extension of the medial edge of the temporal lobe. The entire hippocampal formation has a length of about 5 cm from its anterior end at the amygdala to its tapering posterior end near the splenium of the corpus callosum.[5]

### Dentate gyrus

The dentate gyrus is composed of three layers: an outer acellular molecular layer, a granular middle layer and an inner polymorphic layer.

### Hippocampus

The word *hippocampus* means “Sea Horse” in Greek. Hippocampus is a trilaminate structure with an outer molecular layer, a middle pyramidal layer and an inner polymorphic layer. On the basis of differences in cytoarchitecture and connectivity, the hippocampus has four fields (named by Lorente de No in 1934): CA1, CA2, CA3 and CA4 (CA: Cornu Ammonis). To embryologic enthusiasts, CA4 is not part of the Cornu Ammonis but is a separate structure—the dentate hilus. The thin layer of fibers adjacent to the polymorphic layer of the hippocampus is known as the *alveus*. These fibres coalesce to form the fimbria and subsequently, the crura of the fornix (main efferent pathway of the hippocampal formation). The crura of the fornix converge to form the body of the fornix, which later forms the columns of the fornix and pass through the hypothalamus into the mammillary bodies.[5,7]

### Subicular complex

The subicular complex has three components: the presubiculum, the parasubiculum and subiculum. Subiculum is the transitional zone between the six-layered, entorhinal cortex and the three-layered hippocampus.[5]

### Amygdala

Identified by Burdach in the early 19^th^ century, amygdala, an almond-shaped structure deep within the temporal lobe, is a collection of nuclei lying beneath the uncus. Lying at the anterior end of the hippocampal formation and the anterior tip of the inferior horn of the lateral ventricle, it merges with the periamygdaloid cortex, which forms part of the surface of the uncus. The amygdaloid complex is structurally diverse and comprises of approximately 13 nuclei. These are further divided into subdivisions that have extensive internuclear and intranuclear connections. The major groups are:

- Basolateral nuclei
- Cortical-like nuclei
- Centromedial nuclei
- Other amygdaloid nuclei
- Extended Amygdala[8] (centromedial amygdala, sublenticular substantia innominata and bed nucleus of the stria terminalis)[9]

### Septal area

The septal area is a gray matter structure, immediately above the anterior commissure, has extensive reciprocal connections with the hippocampus (via the fornix). The septal area also projects to the habenula nuclei via the stria medullaris thalami and the anterior hypothalamus.[5]

### Hypothalamus

The hypothalamus lies at the center of the limbic system and is at the confluence of many neural pathways. It is subdivided from anterior to posterior into three zones: the supraoptic region, the tuberal region and the mammillary region. The three zones are divided on each side into medial and lateral areas by the fornix. The hypothalamic nuclei include the following [Table 1].[5]

**Table 1:** Hypothalamic nuclei

Region	Medial area	Lateral area
Supraoptic	Supraoptic nucleus	Lateral nucleus
	Paraventricular nucleus	Part of Supraoptic nucleus
	Anterior nucleus	
	Suprachiasmatic nucleus	
Tuberal	Dorsomedial nucleus	Lateral nucleus
	Ventomedial nucleus	Lateral tuberal nuclei
	Arcuate nucleus	
Mamillary	Mamillary body Posterior nucleus	Lateral nucleus

## FUNCTIONAL CIRCUITRY

### Papez circuit

**Figure 1:**
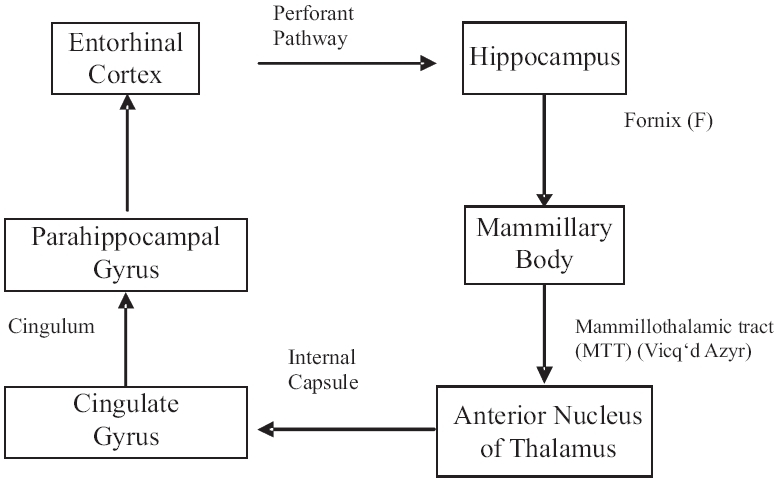
Papez circuit

James Papez's delineation of a circuit after injecting rabies virus into a cat's hippocampus and monitoring its progression through the brain, unraveled the basis of cortical control of emotion. Further elaboration of the circuit has included the prefrontal cortex (PFC), amygdala and septum among other areas [Figure 1].[1,2]

### Hippocampal afferents

The major input to the hippocampal formation arises from neurons in layers II and III of the entorhinal cortex. In addition, some septal and hypothalamic fibres reach the hippocampal formation via the fornix. A few fibres also arrive from the contralateral hippocampal formation via the hippocampal commissure.[10]

### Internal circuit

The intrinsic connections of the hippocampus involve fibers from the entorhinal area, dentate gyrus, Ammon's horn and the subiculum. The three primary pathways of this area are called the perforant pathway, mossy fibers and Schaffer collaterals. The existence of a fourth pathway, the alvear path from the entorhinal area to Ammon's horn through the alveus, has been questioned.

**Figure 2:**
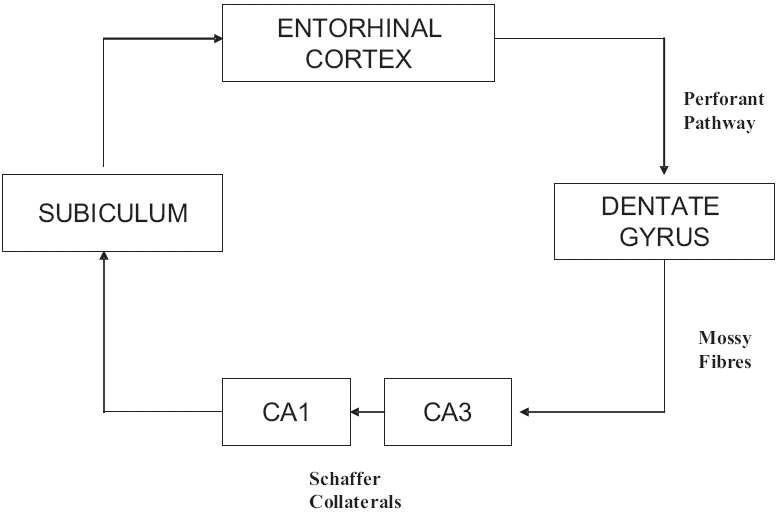
Internal circuit of hippocampal formation

The perforant path is considered the main afferent pathway to the hippocampus, where glutamatergic fibers from the entorhinal area “perforate” the subiculum and reach the dentate gyrus (granule cell layer), traversing the fused hippocampal fissure. Investigators debate whether some perforant fibers reach Ammon's horn. The glutamatergic mossy fibers then extend from the dentate gyrus to CA3 (pyramidal layer), although some efferent fibers from CA3 project to the fimbria. Many axons of CA3, however, give off the Schaffer collaterals that reach the dendrites of CA1. CA1 is considered the main output of the hippocampus with fibers extending to the alveus, fimbria and then fornix. A supplementary linkage with the subiculum also is believed to be present [Figure 2].[10]

### Hippocampal efferents

**Figure 3:**
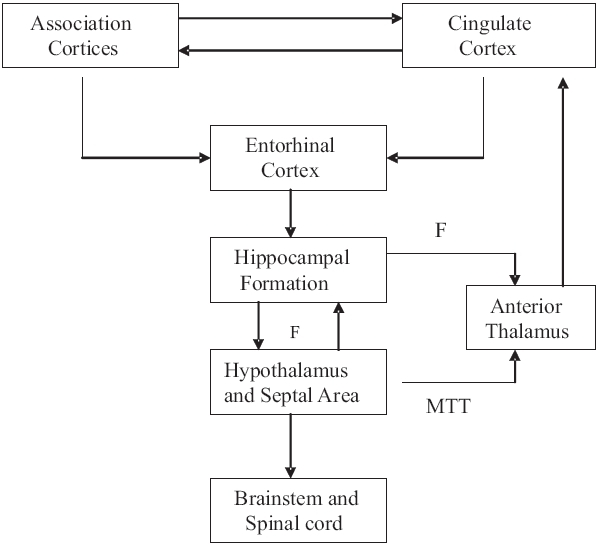
Connections of the hippocampal formation (F: Fornix, MTT: Mammilothalamic tract)

The efferent fibers from the hippocampal region form three groups: precommissural fornix, postcommissural fornix and nonfornical fibers. The precommissural fibers of the fornix may originate from the cornu ammonis or the subiculum. These fibers travel within the fimbria, crura and body of the fornix. The cornu ammonis fibers terminate exclusively in the lateral septal nucleus, whereas the subicular fibers are distributed to nucleus accumbens, anterior olfactory nucleus, lateral septal nucleus, precommissural hippocampus, medial frontal cortex and gyrus rectus. The postcommissural fibers mostly terminate in the mamillary body, although some fibers also project to the anterior thalamic nucleus, bed nucleus of the stria terminalis and ventromedial hypothalamic nucleus. The nonfornical fibers project directly from the hippocampus to the entorhinal area as well as to the posterior cingulate and retrosplenial cortices and the amygdala [Figure 3].[10]

### Circuits of amygdala

**Figure 4:**
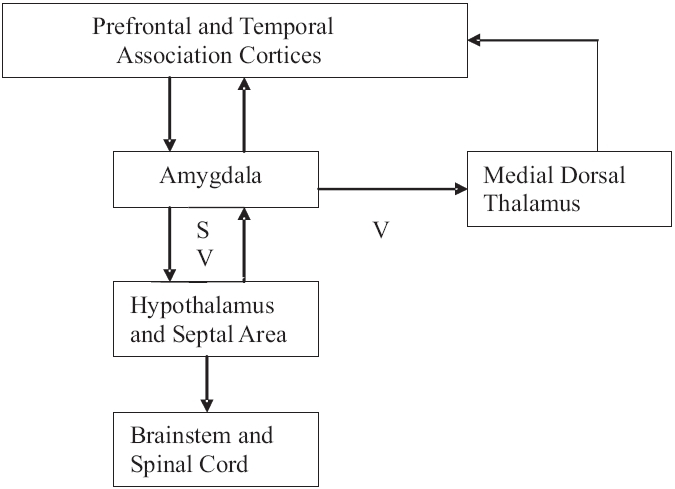
Amygdalo-septal pathway, S: Stria terminalis, V: Ventral amygdalofugal pathway

Amygdala serves to integrate information processing between prefrontal / temporal association cortices and the hypothalamus. The amygdala has two major output pathways:

- Dorsal route via stria terminalis projects to the septal area and hypothalamus.
- Ventral route via the ventral amygdalofugal pathway terminates in the septal area, hypothalamus and the medial dorsal thalamic nucleus [Figure 4].

**Figure 5:**
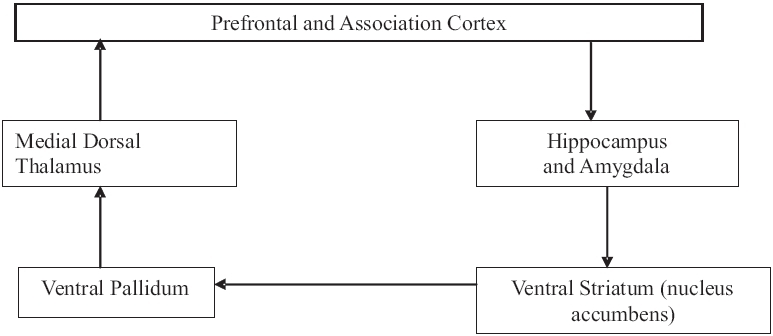
Amygdala basal ganglia circuit

The amygdala has also got connections with the basal ganglia circuit via its projections to the ventral pallidum and ventral striatum, which is relayed back to the cortex via the dorsomedial nucleus of the thalamus [Figure 5].

### Basolateral circuit

**Figure 6:**
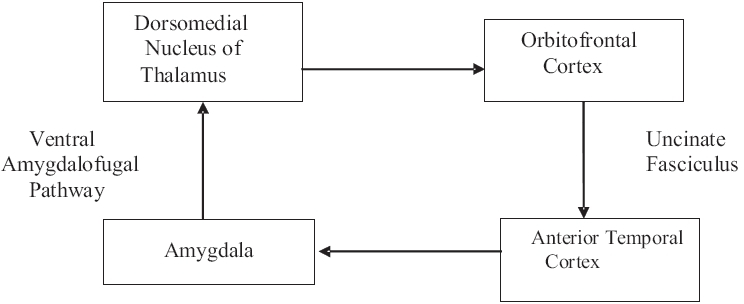
Basolateral circuit

This circuit is relayed via the basolateral amygdala. This circuit consists of the orbitofrontal and anterior temporal cortex, amygdala (especially the basolateral amygdala) and magnocellular division of the dorsomedial nucleus of the thalamus (frontothalamic pathway), which relays back to the orbitofrontal cortex.[11] This circuit encodes information about social signals and social plans for social acts. The circuit has been proposed as a substrate for the human ability to infer the intentions of others from their language, gaze and gestures (Theory of mind and social cognition)[Figure 6].[12]

## FUNCTIONS OF THE LIMBIC SYSTEM

The functions of the various structures of limbic system are outlined below [Table 2].

**Table 2:** Functions of individual structures and connections

Areas	Functions
Cingulate gyrus	Autonomic functions regulating heart rate and blood pressure as well as cognitive, attentional and emotional processing.
Parahippocampal gyrus	Spatial memory
Hippocampus	Long-term memory
Amygdala	Anxiety, aggression, fear conditioning; emotional memory and social cognition.
Hypothalamus	Regulates the autonomic nervous system via hormone production and release. Secondarily affects and regulates blood pressure, heart rate, hunger, thirst, sexual arousal and the circadian rhythm sleep/wake cycle.
Mammilary body	Memory
Nucleus accumbens	Reward, Addiction

### Olfaction

**Figure 7:**
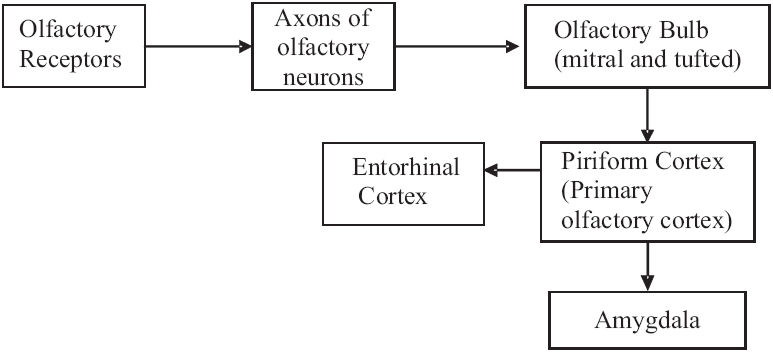
Olfactory role of limbic system

The limbic structures are closely related to the olfactory cortex and have a role in the processing of olfactory sensation. Amygdala is involved in the emotional response to smell while another limbic structure—the entorhinal cortex, is concerned with olfactory memories [Figure 7].[13]

### Appetite and eating behaviors

Amygdala plays a role in food choice and emotional modulation of food intake. The lateral nucleus of the hypothalamus is the center for control of feeding whereas the ventromedial nucleus functions as the satiety center.[14]

### Sleep and dreams

Positron emission tomography (PET) and functional magnetic resonance imaging (fMRI) have shown that the limbic system is one of the most active brain areas during the process of dreaming. The limbic system probably interweaves unconscious primal emotions with our conscious cognitive thoughts and perceptions and thereby ties together emotions and memory during rapid eye movement (REM) sleep to form the content of dreams. The suprachiasmatic nucleus of hypothalamus is the circadian rhythm generator controlling the sleep-wake cycle.[15] The ventrolateral preoptic nucleus (VLPO) of the hypothalamus sends projections to the histaminergic tuberomamillary nucleus (TMN), the serotonergic dorsal and median raphe nucleus and the noradenergic locus coeruleus. It also sends axons that terminate within the cholinergic basal forebrain, the pedunculopontinethalamic nucleus (PPT) and lateral dorsal thalamic nucleus (LDT). The VLPO projections to these areas are inhibitory in nature as they are γ-aminobutyric acid-ergic (GABAergic) and galaninergic. The VLPO via its inhibition of the major arousal mechanisms, functions as a ‘sleep switch’, promoting sleep. Its reciprocal relationship with the major arousal areas helps it to function as one half of a ‘flip-flop’ circuit, the stable firing (flip-flop) of which prevents intermediate states of sleep and wakefulness. The VLPO by its disinhibition of the PPT-LDT also promotes REM sleep. The lateral hypothalamic area (LHA) contains orexinergic neurons that promote wakefulness. The orexinergic neurons inhibit the sleep-promoting VLPO and the REM sleep-promoting neurons in the PPT-LDT. The orexinergic neurons also increase the firing of the locus coeruleus, dorsal raphe and the TMN and in a way, act as a finger pressing the 'flip-flop' circuit switch into the wakefulness position.[16]

### Emotional responses

#### Fear

Fear responses are produced by the stimulation of the hypothalamus and amygdala. Amygdalar destruction abolishes fear and its autonomic and endocrine responses. Amygdala is also involved in fear learning, which is blocked when long-term potentiation (LTP) is disrupted in pathways to the amygdala. Imaging studies have shown that viewing fearful faces activates the left amygdala.[14,17]

#### Rage and placidity

Rage responses to minor stimuli are observed after removal of the neocortex. The destruction of the ventromedial hypothalamic nuclei and septal nuclei in animals with intact cerebral cortices may induce rage. Rage may also be generated by the stimulation of an area extending back through the lateral hypothalamus to the central gray matter of the midbrain. Bilateral destruction of the amygdala results in placidity. However, when the ventromedian nucleus is destroyed after the destruction of the amygdala, the placidity generated is converted to rage.[14]

#### Autonomic and endocrine responses to emotion

**Figure 8:**
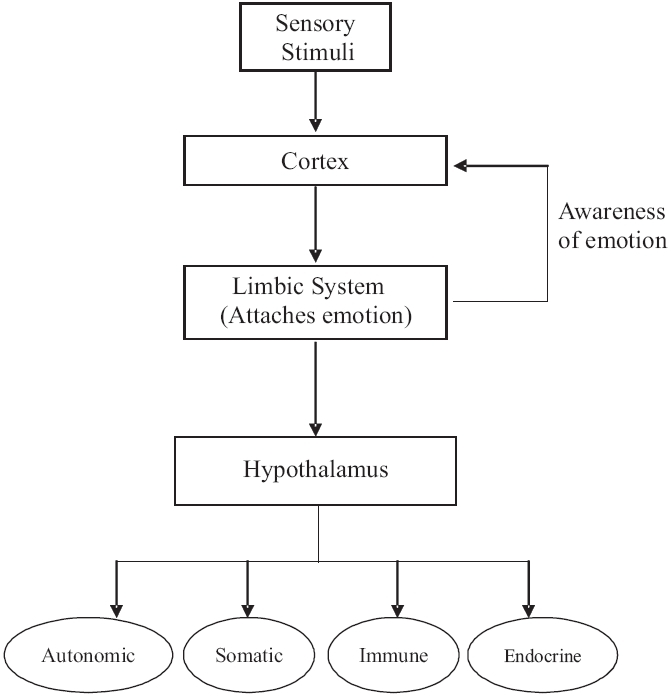
Limbic system and autonomic and endocrine responses

Limbic stimulation causes changes in respiration and blood pressure. The stimulation of the cingulate gyrus and hypothalamus can elicit autonomic responses. There is however little evidence for localization of autonomic responses in limbic circuitry.[14] Hypothalamic autonomic responses are triggered by a complex phenomenon mediated by the cortical and limbic structures processing drives and emotions. The fear and rage responses mediated by the limbic system cause stimulation of various parts of the hypothalamus, especially the lateral areas and produce diffuse sympathetic discharge. The massive sympathetic discharge during stress is called the “flight or fright response”. Stress via cortical and limbic connections causes release of corticotropin-releasing hormone (CRH) from the paraventricular nuclei of the hypothalamus. CRH release mediates endocrine and immune responses [Figure 8].[18]

### Sexual behavior

**Figure 9:**
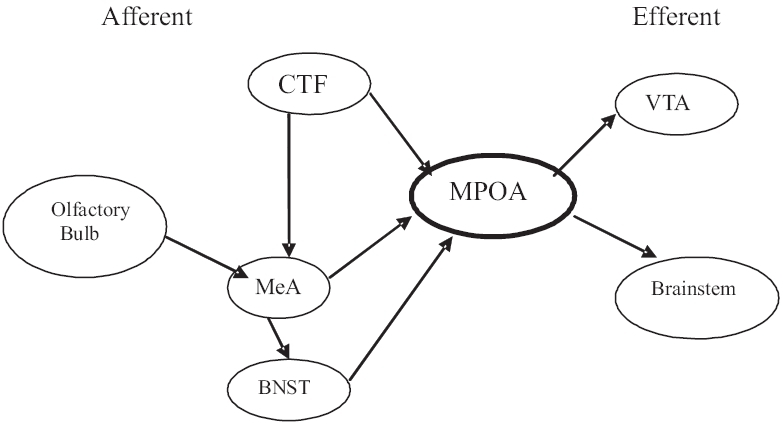
Neural Circuit of Male Sexual Behavior CTF: Central Tegmental Field, MeA: Medial Amygdala, BNST: Bed Nucleus of Stria Terminalis, VTA: Ventral Tegmental Area, MPOA: Medial Preoptic Area

The medial preoptic area of the hypothalamus is a key structure in the central control of male sexual behavior. Chemosensory efferents from the main and accessory olfactory systems project to the medial amygdala (MeA). MeA sends direct and indirect innervations (through the bed nucleus of the stria terminalis) to the medial preoptic area (MPOA). MPOA and MeA receive genitosensory input from the spinal cord through the central tegmental field (CTF). The parvocellular portion of the CTF called the subparafascicular nucleus (SPFp) seems to be especially important for stimuli related to ejaculation. The MPOA sends efferents to the paraventricular nucleus of the hypothalamus (PVN), the ventral tegmental area, the nucleus paragigantocellularis and other autonomic and somatomotor areas [Figure 9].[19]

The parvocellular part of the paraventricular nucleus (PVN) of the hypothalamus contains neurons that send direct oxytocinergic and vasopressinergic projections to the lumbosacral cord. Dopamine can trigger penile erection by acting on oxytocinergic neurons located in the paraventricular nucleus of the hypothalamus. Activation of oxytocinergic neurons originating in the PVN and projecting to extrahypothalamic brain areas, by dopamine and its agonists—excitatory amino acids (N-methyl-D-aspartic acid) or oxytocin itself or by electrical stimulation leads to penile erection. The inhibition of these neurons on the other hand, by GABA and its agonists or by opioid peptides and opiate-like drugs, inhibits this sexual response. The activation of these neurons is secondary to the activation of nitric oxide synthase (NOS), which produces nitric oxide. At least some of the glutamatergic inputs to the MPOA are from the medial amygdala (MeA) and bed nucleus of the stria terminalis (BNST), which mediate the female-stimulated increase in dopamine, which in turn, enhances copulatory ability. Extracellular glutamate in the MPOA increases during copulation, especially during ejaculation and increased glutamate facilitates copulation and genital reflexes.[20]

### Addiction and motivation

**Figure 10:**
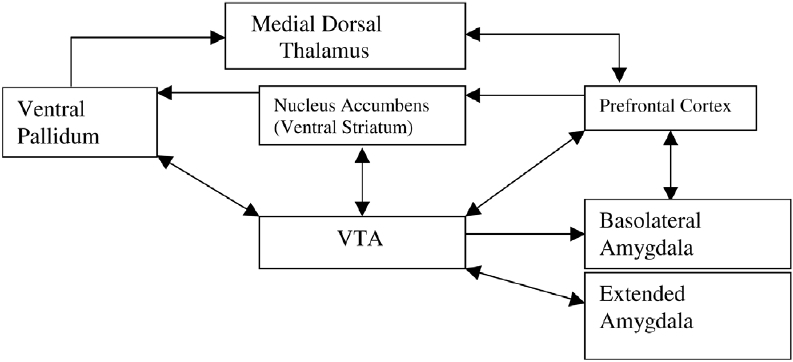
Pathway for motivated or goal directed behavior^(modified from Kalivas and Volkow)^[21]

The reward circuitry underlying addictive behavior includes amygdala and nucleus accumbens. The amygdala plays a central role in cue-induced relapse. Relapse associated with cues, stress and a single dose of a drug of abuse (Comment: which one? What kind?) results in release of excitatory neurotransmitters in brain areas like hippocampus and amygdala. The pathway of motivated behavior involves the prefrontal cortex, the ventral tegmental area (VTA), the amygdala especially the basolateral amygdala and extended amygdala, the nucleus accumbens core and the ventral pallidum. This pathway is involved in the motivation to take drugs of abuse (drug-seeking) and the compulsive nature of drug-taking [Figure 10].[21]

### Memory

#### Emotional memory

Emotion has powerful influence on learning and memory. Amygdala, in conjunction with prefrontal cortex and medial temporal lobe, is involved in consolidation and retrieval of emotional memories. Amygdala, prefrontal cortex and hippocampus are also involved in the acquisition, extinction and recovery of fears to cues and contexts. Hippocampus is critical for long-term, declarative memory storage.[22]

#### Medial temporal lobe memory system

The components include the hippocampus and adjacent cortex, the parahippocampal regions (PHG) and the entorhinal and perirhinal regions. This memory system is involved in the storage of new memories.[23]

#### Diencephalic memory system

The diencephalic memory circuit consists of the hypothalamus, mammillary body and the dorsomedial nucleus of thalamus. This circuit is important for the storage of recent memory; a dysfunction of this circuit results in Korsakoff's syndrome.[23]

### Social cognition

Social cognition refers to thought processes involved in understanding and dealing with other people. Social cognition involves regions that mediate face perception, emotional processing; theory of mind (TOM); self-reference and working memory. Together, the functioning of these regions would support the complex behaviors necessary for social interactions. Limbic structures involved are the cingulate gyrus and amygdala.[12]

## CLINICAL IMPLICATIONS

### Epilepsy

Temporal lobe epilepsy is the most common epilepsy in adults and is most often caused by hippocampal sclerosis. Hippocampal sclerosis with additional involvement of the amygdala and parahippocampal gyrus is termed mesial temporal sclerosis (MTS). CA1 or the Sommer sector, is the region most vulnerable to hypoxia. CA4, sometimes described as the endfolium, has intermediate vulnerability to insults, whereas CA3, which enters the concavity of the gyrus dentate, is only slightly vulnerable. CA2 is the most resistant and well-preserved sector. The frequency and widespread distribution of these cerebral abnormalities suggest that MTS is not limited to the medial temporal lobe but instead, represents a limbic system disorder.[24]

### Limbic encephalitis

Limbic encephalitis is a paraneoplastic syndrome that has been reported with carcinoma of the lung, breast and some other primaries. The mechanism of disease is not known but it manifests as encephalitis that primarily involves the hippocampus, amygdala, cingulate gyrus, insula and orbital-frontal cortex. Afflicted patients develop subacute onset of memory loss, dementia, involuntary movements and ataxia.[25]

### Dementia

Degenerative changes in the limbic system likely have a role in the genesis of neurodegenerative diseases, particularly Pick's disease and Alzheimer's disease. Marked atrophy is found in the limbic system, most notably the dentate gyrus and hippocampus. In Alzheimer's disease, senile plaques and neurofibrillary tangles are dispersed throughout the cerebral cortex and basal ganglia, but the hippocampus and amygdala are often severely involved.[26]

### Anxiety disorders

Anxiety disorders may be the result of a failure of the anterior cingulate and hippocampus to modulate the activity of the amygdala (top-down regulation). A fear circuitry, involving the amygdala, prefrontal and anterior cingulate has been described (bottoms-up regulation).[27]

### Schizophrenia

Studies have shown reduced limbic volumes in schizophrenia. The Papez circuit is probably involved in schizophrenia. The evidence for this is the distortion of cortical neuronal organization of layer II of the ERC, decreased size of hippocampus and the reduced number of GABAergic cells in the cingulate and anterior thalamus with resultant glutamatergic excitotoxicity. The other circuit involved is the basolateral circuit which mediates the social cognition deficits in schizophrenia.[28]

### Affective disorders

**Figure 11:**
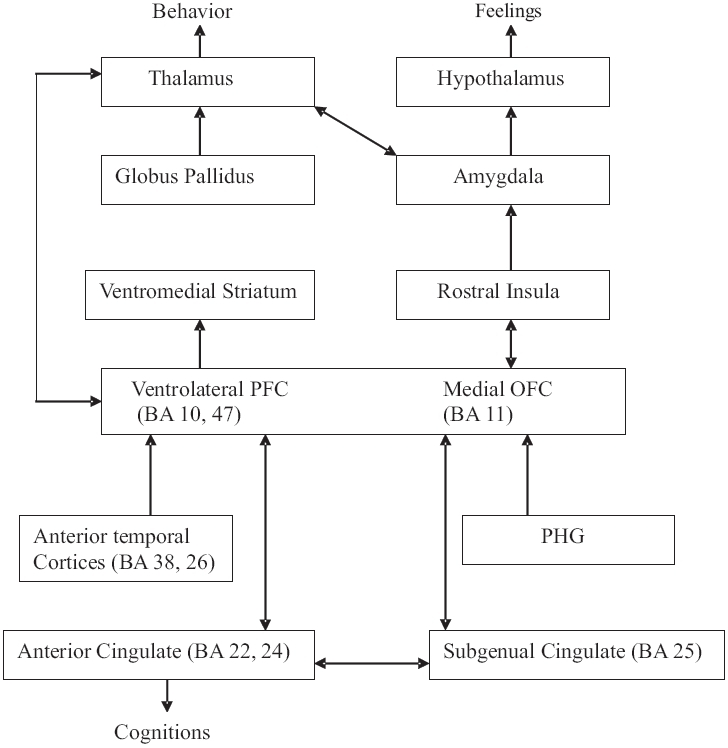
Anterior Limbic Network: PFC: Prefrontal Cortex, PHG: Parahippocampal Gyrus, BA: Broadman area (adapted from Strakowsky *et al*.[30])

Studies have shown variation in the volumes of the frontal lobes, basal ganglia, amygdala and hippocampus in affective disorders. Functional studies have revealed decreased prefrontal and anterior cingulate activity in affective disorders. The anterior cingulate is the center for integration of attentional and emotional output and helps effortful control of emotional arousal.[29] Recently, researchers have posited that this spectrum of affective and cognitive symptomatology represents dysfunction within a single extended network—the anterior limbic network, which includes prefrontal regions and subcortical structures such as the thalamus, striatum and the amygdala. The dysfunction of this system (anterior limbic network) is suggested in bipolar disorder, but its role in depression is unclear [Figure 11].[30]

### ADHD

Limbic structures have been implicated in the genesis of attention deficit / hyperactivity disorder (ADHD). The enlarged hippocampus in children and adolescents with ADHD may represent a compensatory response to the presence of disturbances in the perception of time, temporal processing and stimulus-seeking associated with ADHD. Disrupted connections between the amygdala and orbitofrontal cortex may contribute to behavioral disinhibition seen in individuals with ADHD.[31]

### Kluver-Bucy syndrome

Kluver-Bucy syndrome results due to a bilateral destruction of the amygdaloid body and inferior temporal cortex. It is characterized by visual agnosia, placidity, hypermetamorphosis, hyperorality and hypersexuality. This disorder may be caused by many conditions including cerebral trauma; infections including herpes and other encephalitides; Alzheimer's disease and other dementias; Niemann-Pick disease and cerebrovascular disease.[14]

### Korsakoff's psychosis

Korsakoff's psychosis is caused by damage to mammillary bodies, dorsomedial nucleus of thalamus and hypothalamus (diencephalic memory circuit). It is a syndrome associated with chronic prominent impairment of recent and remote memory. Recent memory is characteristically more disturbed than remote memory. Immediate recall is usually preserved. Confabulation may be marked but is not invariably present.[20]

### Autism

Autism and Asperger's syndrome involve the disproportionate impairment in specific aspects of social cognition. Limbic structures involved include the cingulate gyrus and amygdala, which mediate cognitive and affective processing. The basolateral circuit integral for social cognition is disrupted in autism spectrum disorders.[28]

## CONCLUSION

The limbic system plays a pivotal role in behavior. The intricate functional neuroanatomy of limbic system with its diverse circuits may explain some of the manifestations of neuropsychiatric disorders. Relentless research has identified the role of the amygdala in various anxiety disorders and emotional memory. The monitoring role of anterior cingulate, the trisynaptic hippocampal circuitry underlying cognitive functioning and the significance of hypothalamus in various neurovegetative functions suggest the integral role of the limbic system in understanding human behavior and its aberrations.
